# Metabolomics in plasma of Malawian children 7 years after surviving severe acute malnutrition: “ChroSAM” a cohort study

**DOI:** 10.1016/j.ebiom.2019.06.041

**Published:** 2019-06-27

**Authors:** Celine Bourdon, Natasha Lelijveld, Debbie Thompson, Prasad S. Dalvi, Gerard Bryan Gonzales, Dominic Wang, Misagh Alipour, Eytan Wine, Emmanuel Chimwezi, Jonathan C. Wells, Marko Kerac, Robert Bandsma, Moffat J. Nyirenda

**Affiliations:** a Department of Translational Medicine, Hospital for Sick Children, Toronto, Canada; b The Childhood Acute Illness & Nutrition Network, Canada; c Centre for Global Child Health, Hospital for Sick Children, Toronto, Canada; d Malawi-Liverpool Wellcome Trust Clinical Research Programme, University of Malawi College of Medicine, Blantyre, Malawi; e Institute for Global Health, University College London, London, UK; f Caribbean Institute for Health Research, University of the West Indies, Kingston, Jamaica; g Morosky College of Health Professions and Sciences, Gannon University, Erie, PA, USA; h Gastroenterology, Department of Internal Medicine and Paediatrics, Ghent University, Ghent, Belgium; i VIB Inflammation Research Centre, Ghent, Belgium; j Division of Pediatric Gastroenterology and Nutrition, University of Alberta, Edmonton, Canada; k Childhood Nutrition Research Centre, Institute of Child Health, University College London, London, UK; l Department of Population Health, London School of Hygiene & Tropical Medicine, London, UK; m Department of Biomedical Sciences, College of Medicine, University of Malawi, Blantyre, Malawi; n Division of Gastroenterology, Hepatology and Nutrition, Hospital for Sick Children, Toronto, Canada; o Department of Pediatrics, University Medical Center Groningen, Groningen, the Netherlands; p Department of Nutritional Sciences, Faculty of Medicine, University of Toronto, Toronto, Canada; q MRC / UVRI Uganda Research Unit, Entebbe, Uganda

## Abstract

**Background:**

More children are now surviving severe acute malnutrition (SAM), but evidence suggests that early-life malnutrition is associated with increased risk of long-term cardio-metabolic disorders. To better understand potential mechanisms, we studied the metabolite profiles of children seven years after treatment for SAM.

**Methods:**

We followed-up children (*n* = 352) treated for SAM in 2006–2007, at Queen Elizabeth Central Hospital, in Malawi. Using nuclear magnetic resonance spectroscopy, tandem mass spectrometry and enzyme-linked immunosorbent assay, we measured circulating metabolites in fasting blood in a subset of SAM survivors (*n* = 69, 9·6 ± 1·6 years), siblings (*n* = 44, 10·5 ± 2·7 years), and age and sex-matched community controls (*n* = 37, 9·4 ± 1·8 years). Data were analysed using univariate and sparse partial least square (sPLS) methods. Differences associated with SAM survival, oedema status, and anthropometry were tested, adjusting for age, sex, HIV, and wealth index.

**Findings:**

Based on 194 measured metabolites, the profiles of SAM survivors were similar to those of siblings and community controls. IGF1, creatinine, and FGF21, had loading values >0·3 and ranked stably in the top 10 distinguishing metabolites, but did not differ between SAM survivors and controls with univariate analysis. Current stunting was associated with IGF1 (β = 15·2, *SE* = 3·5, partial R^2^ = 12%, *p* < 0·0001) and this relationship could be influenced by early childhood SAM (β = 17·4, *SE* *=* 7·7, partial R^2^ = 2·8%, *p* = 0·025). No metabolites were associated with oedema status, duration of hospital stay, anthropometry measured during hospitalization, nor with changes in anthropometry since hospitalization.

**Interpretation:**

In this group of survivors, SAM was not associated with longer-term global metabolic changes 7 years after treatment. However, SAM may influence the relationship between current stunting and IGF1. Further risk markers for NCDs in SAM survivors may only be revealed by direct metabolic challenge or later in life.

**Research in context**

**Evidence before this study**

Following the concept of “developmental origin of health and disease” (DOHaD), evidence from studies of prenatal malnutrition suggests that early-life insults can increase long-term risk of disease. With more children surviving episodes of severe acute malnutrition (SAM), there is growing concern for the longer-term consequences. However, few studies have followed-up survivors of SAM, especially beyond 1 year post-treatment, as described in our 2016 publication of growth and functional outcomes for this Malawi cohort (“ChroSAM study”). Previously, we showed that Malawian survivors of SAM have a “thrifty” growth trajectory, reduced lean mass levels, and reduced muscle strength, seven years after treatment. A small number of studies have looked at metabolism of SAM children during treatment and found hepatic steatosis, abnormal glucose homeostasis, and changes in several classes of metabolites or proteins, including acylcarnitines, inflammatory cytokines, fatty acids, amino acids, sphingolipids and hormones related to appetite and energy metabolism. Some of these changes have been associated with the development of type 2 diabetes 12 years prior to onset and a number of sphingolipids have been linked to insulin resistance and metabolic syndrome. However, it is not currently known whether metabolic disturbances seen during SAM and immediate recovery persist beyond treatment.

**Added value of this study**

We report the first quantitative metabolomics study of long-term SAM survivors and show that 7 years after treatment, metabolic profiles of SAM survivors are similar to those of sibling and community controls. We also found that measures of stunting in largely prepubescent children were associated with low IGF1 and, having experienced childhood SAM modulated this relationship.

**Implications of all the available evidence**

The evidence therefore suggests that SAM has long-term implications for growth, body composition and muscle strength, and stunted SAM survivors have even lower IGF1 levels than other stunted children from similar low socioeconomic communities. These outcomes are all associated with greater non-communicable disease (NCD) risk in later life. However, without evidence of metabolic profile changes or other early signs of NCD development, we do not know whether SAM survivors have been subject to epigenetic changes, as is the case for low birth weight survivors, nor whether they are at greater risk of NCDs if their metabolic “load” remains low (i.e. they remain short and thin). Future research is needed, such as comparing SAM survivors to healthier and wealthier children in the same contexts, and assessing the response of SAM survivors to metabolic challenges. Public health programmers and policy makers need to be mindful of the potential long-term consequences of SAM; survival alone is no longer sufficient for affected children.

Alt-text: Unlabelled Box

## 1 Introduction

Severe acute malnutrition (SAM) affects approximately 18 million children under the age of 5 years and remains a significant contributor to mortality, resulting in at least 500,000 deaths/year [1]. Currently, program protocols are focused on the urgent challenge of preventing mortality through the use of ready-to-use therapeutic foods (RUTF) for treatment of SAM and this effort has been largely successful [2,3]. However, with more children surviving episodes of SAM, there is growing concern for the longer-term consequences. Following the concept of “developmental origin of health and disease” (DOHaD), increasing evidence suggests that early-life insults can increase long term risk of disease. For example, foetal undernutrition, manifested as low birth weight, has been associated with increased rates of type 2 diabetes, hypertension and related complications, such as coronary heart disease and stroke in adulthood [[4], [5], [6]]. The mechanisms involved are not fully understood, but environmental exposures could alter development by driving changes in epigenetic marks or organ structure resulting in future physiological consequences [4].

The length of the “window of plasticity” for when insults can occur and cause these epigenetic changes is not yet clear. There is strong evidence for insults in utero, but less evidence for the long-term effects of insults in infancy, such as SAM. However, acute post-natal malnutrition has been associated with profound metabolic disturbances such as hepatic steatosis and abnormal glucose homeostasis [[7], [8], [9]]. SAM, both during the acute episode or during the immediate recovery phase, is associated with changes in several classes of metabolites or proteins, including acylcarnitines, inflammatory cytokines, fatty acids, amino acids, sphingolipids and hormones related to appetite and energy metabolism [10,11]. Some of these changes, such as increased branched chain amino acids, have been associated with the development of type 2 diabetes 12 years prior to onset [12] and a number of sphingolipids (particularly ceramides) have been linked to insulin resistance and metabolic syndrome [13].

Previously, we showed that Malawian survivors of SAM showed a “thrifty” growth trajectory and reduced lean mass levels, seven years after treatment. These phenotypic changes are similar to those described in low birth weight children and are associated with future cardiovascular and metabolic disease [14]. Taken together, this evidence suggests that SAM may induce metabolic changes linked to long-term risk of NCDs. However, the extent to which these SAM-related changes persist or whether they are mechanistically linked to NCDs is unknown. Also, it remains unclear how the severity, duration and clinical phenotype of the SAM episode (oedematous versus severe wasting) influence the cardio-metabolic risk profile subsequent to severe malnutrition.

This study aimed to better understand the longer-term metabolic consequences of SAM and identify early markers that could inform preventive interventions. Using a large-scale targeted metabolomics approach with a focus on glucose and hepatic metabolism previously associated with NCDs, we characterized the profiles of SAM survivors from the “ChroSAM cohort” [14] seven years post-discharge and compared them to community and sibling controls.

## 2 Materials and methods

### 2.1 Study design and participants

As detailed in the flow diagram (Supplemental Fig. 1), an initial cohort of 1024 patients were admitted for treatment of SAM between July 2006 and March 2007 at Queen Elizabeth Central Hospital in Blantyre, Malawi. Median age at hospitalization was 21·5 months (IQR 15–32). At that time, SAM was defined as weight-for-height (WHZ) < 70% of the median (NCHS reference), or mid-upper arm circumference (MUAC) < 11 cm, or presence of nutritional oedema [15]. Patients were followed-up after seven years (*n* = 352); 217 siblings and 184 age-and-sex matched community controls were also recruited [14]. Siblings were defined as being closest in age to the case child, age 4–15·9 years with no history of SAM. Community controls were recruited randomly by spinning a bottle at the case child's house and going door to door in that direction [16]. Eligibility criteria were: living in the same community, of the same sex, aged within 12 months of the case child and with no history of SAM. Not all SAM survivors had eligible sibling and community controls. Written informed consent was obtained from the child's guardian and additional assent was sought from children older than 13 years.

### 2.2 Ethics statement

Ethical approval for the study was granted by the Malawi College of Medicine Research and Ethics Committee (reference P.02/13/1342), the Hospital for Sick Children Research Ethics Board (reference1000049242) and the University College London Research Ethics Committee (reference 4683/001).

### 2.3 Clinical data

Characteristics of the children with SAM at initial hospital admission are presented in Supplemental Table 1. WHZ, height-for-age (HAZ), and weight-for-age (WAZ) z-scores were calculated using WHO 2006 growth standards for children under 5 years of age or the 2007 standards for those over 5. Anthropometric z-scores at initial hospitalization were calculated using the minimum weight recorded during admission, i.e., when oedema was sufficiently regressed. HIV reactivity or exposure in children <18 months of age was obtained by rapid antibody testing and a broad panel of supplementary clinical variable was also recorded.

### 2.4 Sample collection and metabolite assay

Venous blood was taken after overnight fasting from consenting participants. Samples were spun within an hour of collection and plasma stored at −80 °C. The Metabolomics Innovation Centre (TMIC) at the University of Alberta, Canada, quantified the metabolites in a random subset of samples using one of three approaches: 1) combined direct injection and liquid chromatography coupled to tandem mass spectrometry (MS/MS) 2) nuclear magnetic resonance (NMR) spectroscopy or 3) Enzyme-linked immunosorbent assay (ELISA).

### 2.5 Combined direct injection and liquid chromatography – tandem mass spectrometry

A combination of direct flow injection and reverse-phase liquid chromatography coupled to tandem mass spectrometry (MS/MS) was performed using the AbsoluteIDQ™ Kit (BIOCRATES Life Sciences AG, Austria). Supplemental Table 2 presents all quantified metabolites that passed standard quality control cut-offs defined as having: 1) a mean CV < 25% across experimental batches, 2) <10% missing values, and 3) a median value greater or equal to the lower limit of quantification in study group.

### 2.6 NMR spectroscopy

NMR spectroscopy was used to identify 24 metabolites. All ^1^H NMR spectra were collected on a 700 MHz Avance III (Bruker) spectrometer, and then processed and analysed using the online Bayesil software package which allows for qualitative and quantitative analysis of NMR spectra.

### 2.7 Specific protein quantification

Two commercial Milliplex® Mag kits were used to measure set panels of adipokine and liver metabolism proteins. The human adipokine magnetic bead panel 2 (cat# HADK2MAG-61 K, Millipore, Darmstadt, Germany) was used to quantify: 1) human nerve growth factor (NGF), 2) interleukin 6 (IL6), 3) insulin, 4) leptin (LEP), 5) interleukin 8 (Il8), 6) hepatocyte growth factor (HGF), 7) C—C motif chemokine ligand 2 (CCL2, a.k.a. MCP − 1), 8) tumor necrosis factor alpha (TNF, a.k.a. TNF-α) and 9) interleukin 1 beta (IL1B). The human liver protein magnetic bead panel (cat# HLPPMAG-57 K, Millipore) was used to measure: 1) alpha fetoprotein (AFP), 2) angiopoietin like 3 (ANGPTL3), 3) angiopoietin like 4 (ANGPTL4), 4) angiopoietin like 6 (ANGPTL6, a.k.a. AGF), 5) hepatocyte growth factor (HGF), 6) fatty acid binding protein 1 (FABP1), 7) fibroblast growth factor 19 (FGF19), 8) fibroblast growth factor 21 (FGF21), and 9) fibroblast growth factor 23 (FGF23). Additional proteins were analysed as per manufacturer's instructions using either standard or competitive ELISA: 1) human growth hormone 1 (GH1, cat# DGH00, R&D Systems, Minneapolis, USA), 2) insulin like growth factor 1 (IGF1, cat# DG100, R&D Systems), 3) retinol binding protein 1 (RBP1, cat# EIA-5202, DRG Diagnostics, Marburg, Germany), 4) total 25-OH vitamin D (25(OH)D, cat# EIA-5396, DRG Diagnostics), 5) vitamin D binding protein (GC, a.k.a. DBP, cat# DVDBP0, R&D Systems).

### 2.8 Statistical analysis

Sample size was constrained by the numbers of patients successfully followed-up and by the budget for sample analysis. Based on mean differences and standard deviations in metabolites (lysine and threonine) at admission in children with kwashiorkor vs wasting [11], our sample size is more than sufficient, with 90% power and 5% significance, to detect differences of approximately 35 μmol/L between the groups. For the primary analysis, metabolite profiles of SAM survivors were compared to those of sibling and community controls. Secondary analyses was conducted within the SAM surviving group only and searched for associations between metabolites and measures of: 1) wasting at initial hospitalization, 2) severity of oedema at admission, 3) post-discharge change in WAZ of SAM survivors and 4) measures of stunting (i.e., HAZ) seven years post discharge. Metabolites and proteins were analysed if above the limit of detection (LOD) in 80% of samples in at least one experimental group; and the remaining concentrations below the LOD were set to half the LOD [17]. Principal component analysis (PCA) was used on log transformed and standardized variables to examine inherent clustering, metabolite correlation and sample outliers. Based on PCA and visual inspection of histograms and residuals, 33 outliers were removed out of 31,800 values (0·1%); most were phosphatidylcholine diacyls from one sample, which drove 57% of the variance in this metabolite class. Measured concentrations obtained from Milliplex and ELISA assays were corrected for technical batch effects using ComBat function as implemented in sva package [18]. Associations between metabolic variables and participant groups were tested with linear models on log transformed variables while adjusting for age (months), sex, HIV status and wealth quintile. Potential confounders were selected a priori based on biological plausibility and baseline differences between the groups. Residuals were inspected and obtained *p*-values were corrected using Benjamini and Hochberg False Discovery Rate (FDR) due to the large number of models. sPLS-DA or sPLS was conducted as implemented by the mixOmics package [17]. These methods analyse multivariate correlations between metabolites, group differences or associations with anthropometric variables while concurrently performing feature selection via ℓ^1^ regularization (LASSO [19]). Penalized regression methods shrink coefficients to zero leaving a subset of top features. For this, metabolites were log transformed and adjusted for age, sex, HIV, and wealth; and standardized residuals were analysed after missing values were imputed using bagImpute in the caret package [20]. There is no critical threshold established to attest significance of PLS components, however, negative Q^2^ values indicate that the component is not predictive while empirical values of at least 0·4 are deemed of acceptable predictive value for biological models [21]. After analysing all participants, sub analyses were done in only cases with oedematous SAM, and those without HIV. Data were analysed using R version 3.4.0 [22] and figures generated with ggplot2 and Inkscape [23].

## 3 Results

### 3.1 Patient characteristics

Metabolite data was available for 150 participants; 69 SAM survivors, 44 siblings and 37 community controls (Supplementary Fig. 1); their characteristics are presented in Table 1. Community controls were more likely to decline hospital assessments, including giving a blood sample, than SAM survivors. 55% were male and none had known underlying congenital conditions. Overall, 21% were HIV positive; but children who experienced early childhood SAM had a significantly higher prevalence (31%) compared to controls (8%, *p* = 0·012). The median age of SAM survivors was 9·1 years [interquartile range (IQR), 8·5–10·2] which did not differ from community controls; however, siblings were older on average (11·5 years, [IQR, 7·5–12·8], p = 0·08). Between hospitalization and 7 year follow-up, SAM survivors showed a mean gain of 2·3 (SD = 1·3) in WAZ; 1·6 (SD = 1·5) in BMI-for-age z-score; and 1·5 (SD = 1·3) in HAZ, however they still have significantly lower anthropometric z-scores than control groups. Puberty onset did not differ between groups where a total of 8 children reported to have reached puberty. Wealth asset quintiles were comparable between groups and specifically the proportion of children in the bottom wealth quintile did not differ. An exploration of anthropometric and functional differences between groups at 1-year and 7-years post-discharge has been published previously [14,24].

**Table 1:** Characteristics of participants in the metabolomics subset.

	Descriptive data		
	SAM survivors	Siblings	Community controls
	n = 69	n = 44	n = 37
Clinical characteristics			
Age, years*	9·1 [8·5–10·2]	11·5 [7·5–12·8]	8·7 [8·3–10·5]
Female, n (%)	30/69 (43%)	22/44 (50%)	15/37 (41%)
HIV positive, n (%)	20/64 (31%)	2/25 (8·0%)	2/25 (8·0%)
Started puberty, n (%)	2/69 (2·9%)	4/44 (9·1%)	2/37 (5·4%)
Weight, kg*	23·7 [21·3–26·2]	29·1 [20·4–36·2]	23·8 [22·1–26·3]
Height, cm*	124 [120–129]	134 [118–145]	126 [121–129]
MUAC, mm*	171 [162–180]	184 [165–205]	174 [166–185]
Weight-for-age^Ϯ^, z-score	−1·44 ± 0·93	−1·34 ± 0·85	−1·08 ± 0·88
Height-for-age, z-score	−1·68 ± 1·2	−1·33 ± 0·98	-1·37 ± 1·0
BMI-for-age, z-score	−0·79 ± 0·98	−0·69 ± 0·66	−0·51 ± 0·84
History of hospital admission (not SAM)	14/69 (20%)	7/44 (16%)	9/37 (24%)

Family characteristics			
Mother alive, n (%)	62/68 (91%)	38/41 (93%)	35/36 (97%)
Mother HIV positive, n (%)	22/53 (42%)	12/33 (36%)	6/31 (19%)
Mother literate, n (%)	41/67 (61%)	25/39 (64%)	26/37 (70%)
Children in family*	3·5 (2·8–4·3)	4 (3–5)	4 (3–4)
Families with children who died	21/68 (31%)	12/41 (29%)	3/36 (8·3%)
Birth order*	2 (1–4)	3 (2–4)	2 (2–4)

Home environment			
Bottom wealth asset quintile, n (%)	10/69 (14%)	5/44 (11%)	4/37 (11%)
Cooking with fire, n (%)	66/69 (96%)	40/41 (98%)	37/37 (100%)
Cooking inside house, n (%)	14/69 (20%)	9/41 (22%)	4/37 (11%)
Unimproved toilet, n (%)	54/69 (78%)	33/41 (80%)	28/37 (76%)

Data are presented as number and percentage, averages and standard deviation or with median and interquartile range as indicated with stars (*). Ϯ Weight-for-age is calculated only in children under 10 years of age. BMI, body mass index; MUAC, mid upper arm circumference; SAM, severe acute malnutrition.

### 3.2 Detection and quantification of metabolites

The median concentrations of all 194 measured metabolites are presented stratified by group in Supplementary Table 2. With targeted MS/MS, 155 different endogenous metabolites were quantified including amino acids (*n* = 18), acylcarnitines (*n* = 26), biogenic amines (*n* = 12), sphingolipids (*n* = 15), and glycerophospholipids (*n* = 84). NMR spectroscopy was used to quantify an additional 24 metabolites classified as: organic acids and derivatives (n = 12); sugars, alcohols and derivatives (*n* = 4), amino acids (*n* = 5), ketone derivatives (n = 1), organic nitrogen compounds (n = 1), carnitines (n = 1). Summary values of amino acids (i.e., total, essential, aromatic, branched chain, glucogenic and ketogenic amino acids) and metabolite ratios such as kynurenine-to-tryptophan and the Fischer ratio were calculated. With the adipokine Milliplex panel, 6 proteins were quantified (i.e., NGF, IL6, LEP, IL8, CCL2, TNF) but IL1B was under detection range in >63% of samples. The liver Milliplex panel quantified 4 proteins (ANGPLT3, ANGPLT4, FGF21 and FGF23) but HGF, FABP1 and FGF19 were under detection range in >38%, 93% and 94% of samples, respectively. ELISA was used to quantify 5 additional molecules: total 25-OH vitamin D and 4 proteins (GH1, IGF1, RBP1, and GC).

### 3.3 Survivors of early childhood SAM were not metabolically distinct from controls

**Fig. 1:**
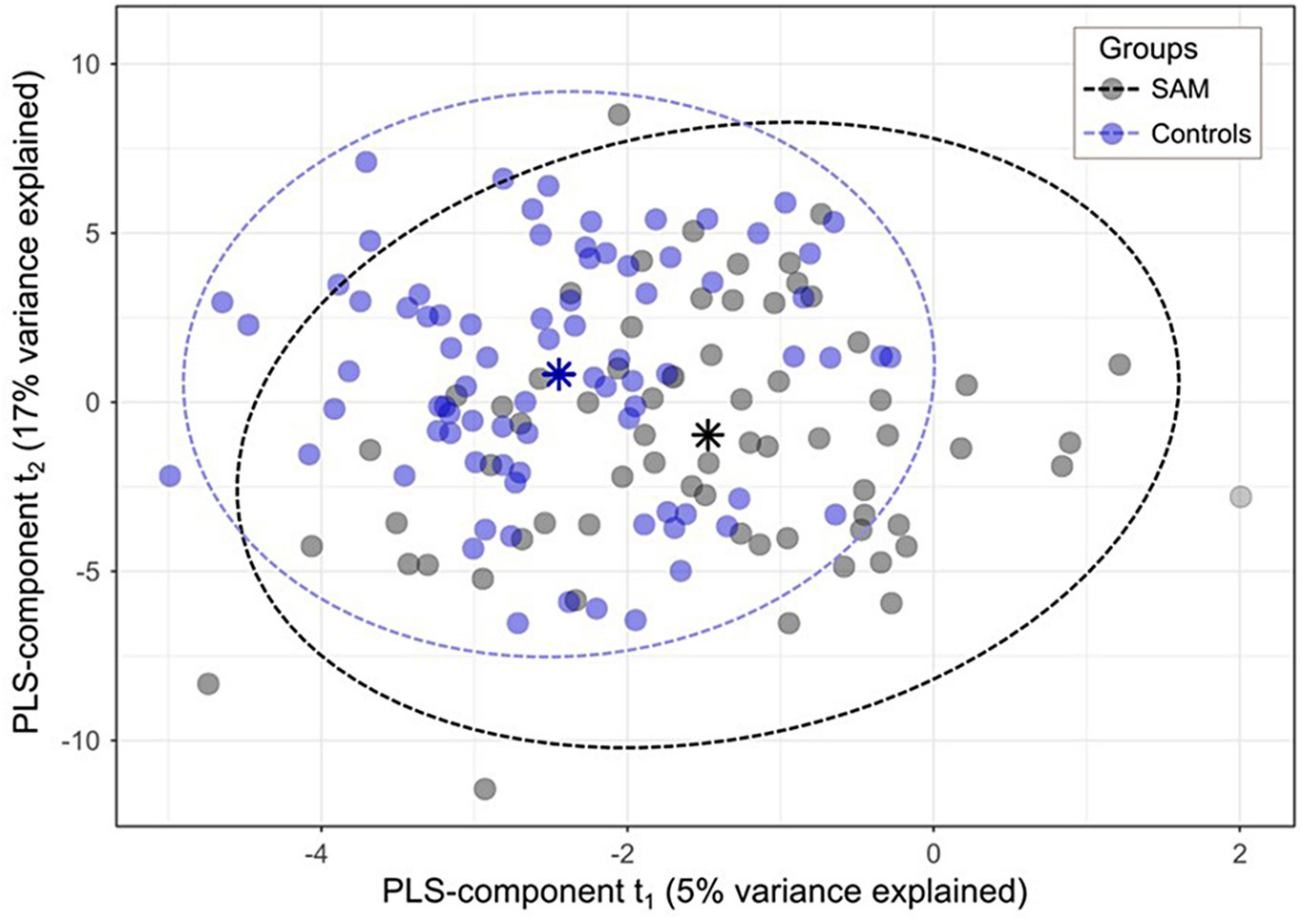
PLS-DA score plot showing clustering of SAM survivors at 7 years post-discharge and controls.

The overall metabolite profile of SAM survivors did not differ from those of either siblings or community controls and results were similar when the control groups were combined. There were no significant differences in concentrations of metabolites when compared using linear regression with *p*-values corrected for FDR (Supplementary Table 2). Using multivariate sPLS-DA, SAM and controls were indistinguishable based on circulating metabolites or proteins and showed a maximum distance balanced classification error rate of 54% (SD, 2·8%) with PLS-component t_1_ explaining 5% of variance (Fig. 1). Table 2 presents the subset of metabolites or proteins that were stably ranked as being most discriminative between SAM and controls by sPLS-DA, i.e., metabolites or proteins selected >80% of the time in the top10 with 10-fold cross validation and showing a PLS-t_1_ loading value >0·3. This table also presents the linear model results for these selected top-ranking features, including IGF1 the top ranked metabolite. These results show that the metabolite and protein profiles of SAM survivors were not distinguishable from those of controls. Sub-analysis including only children that experienced oedematous SAM or those without HIV showed similar results. Supplementary Table 2 presents all the linear models testing for differences between groups.

**Table 2:** Concentrations of top-ranked metabolites selected by sPLS-DA as most different between SAM survivors and controls with associated results from linear regressions.

	SAM *n* = 69	Siblings *n* = 44	Community *n* = 37	Stability^1^	Loading^2^	Group differences^3^							
						SAM vs community				SAM vs siblings			
						β	*SE*	*p*	FDR-*p*	β	*SE*	*p*	FDR-*p*
IGF1, ng/mL	89 [65–110]	120 [86–180]	100 [67–120]	0·9	−0·52	0·16	0·09	0·08	0·98	0·21	0·09	0·018	0·98
Creatinine, μmol/L	30 [16–43]	25 [18–38]	21 [12−32]	0·8	−0·53	−0·26	0·16	0·11	0·98	0·063	0·16	0·7	0·98
FGF21, ng/mL	0·15 [0·06–0·23]	0·1 [0·04–0·2]	0·09 [0·033–0·13]	0·8	0·33	−0·77	0·28	0·008	0·98	−0·32	0·29	0·27	0·98

Data are presented as median [IQR]. sPLS-DA was conducted on standardized log-transformed variables adjusted for age, sex, HIV and wealth. Metabolites were selected when ranked in the top 10 at least 80% of the time across folds and having a > 0.3 PLS-component t_1_ loading value. Feature stability^1^ indicates the frequency at which the metabolite is selected in the top-10 with sPLS-DA across 10-fold cross-validation. The loading^2^ value indicates the correlation strength of the metabolite with PLS-component t_1_. Group differences^3^ were tested using linear models on log transformed variables while adjusting for age, sex, HIV and wealth. Multiple testing correction was done based on all metabolites analysed using Benjamini & Hochberg false discovery rate (FDR). Significance threshold: FDR-corrected *p*-values <0·05. sPLS-DA; sparse partial least square discriminant analysis.

### 3.4 Circulating metabolites or proteins in SAM survivors were not associated with clinical characteristics at hospital admission nor with indicators of anthropometric recovery since hospitalization

Metabolites or proteins were not associated with severity of SAM experience by survivors, i.e. lowest WHZ or WAZ during admission (Supplemental Table 2). None of the metabolites or proteins showed a relationship with duration of hospital stay or clinical phenotype of SAM, i.e., having had severe wasting versus oedematous SAM, nor with the severity of initial oedema at admission. Furthermore, circulating metabolites or proteins were not related to anthropometric recovery in SAM survivors, i.e., with change in WAZ or BMI-for-age z-scores between hospitalization and 7 year follow-up, nor with children categorized as above or below the median anthropometric change (2·5 z-scores) since hospitalization. Significant associations were only found with known confounders such as age, sex, HIV and wealth.

### 3.5 IGF1 was moderately associated with current stunting and this relationship may be influenced by having survived childhood SAM

**Fig. 2:**
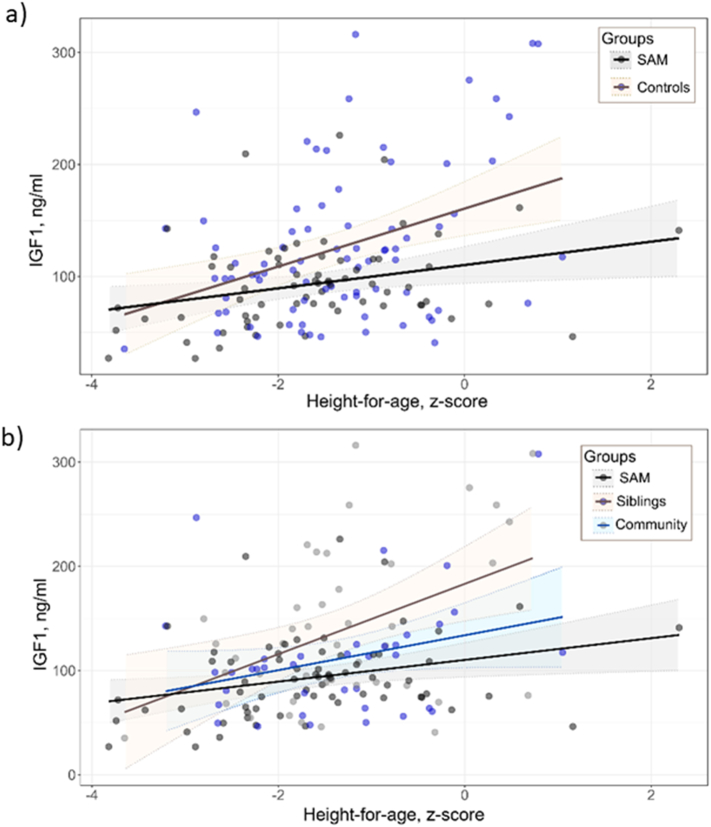
Correlation between stunting and IGF1 levels in children that survived early childhood SAM compared to controls.

Based on sPLS, the overall metabolite profiles were not predictive of current measures of stunting; (Q^2^, a measure of the external validity of the model = 0·002). However, IGF1 again ranked as the top metabolite correlating with current stunting, accounting for 20% of the variance in metabolites or proteins (Table 3) (loading value >0·5 on PLS-component t_1_). Since IGF1 was top ranked and related to both stunting and having experienced early childhood SAM, we further explored this relationship using multivariate linear regression (Table 4 and Fig. 2). Circulating levels of IGF1 showed a partial positive correlation with HAZ of children at the time of ChroSAM (*r* = 0·35, R^2^ = 0·12, *p* < 0·0001) while adjusting for age, sex, HIV and wealth. Although model fit was improved when SAM vs. controls were included (Model 1 vs. Model 2, *p* = 0·025), the partial correlation between IGF1 and SAM survival was weak and the variance explained was small (r = 0·19, R^2^ = 3·2%, p = 0·028). There was no evidence of interaction between stunting and having survived early childhood SAM but survivors do have lower circulating IGF1 levels. There was a significant age and sex interaction, with girls showing a greater positive slope between HAZ and IGF1 (p < 0·005) which could be due to earlier puberty in girls influencing these relationships. However, incorporating self-reported puberty did not improve model fit. Circulating levels of IGF1 were below median levels of European pre-pubertal children with idiopathic short stature (92 ng/mL) [25] in 44% of children in the cohort and this was more common in SAM survivors then in controls (53% vs. 36%, *p* = 0·047). With univariate analysis (Supplemental Table 1), no other metabolite showed a significant relationship with stunting.

**Table 3:** Metabolite concentrations of top-ranked variables related to current stunting as selected by sPLS with associated results from linear regressions.

		SAM	Siblings	Community	Stability^1^	Loading^2^	Height-for-age, z-score		
		n = 69	n = 44	n = 37			β	*SE*	FDR-*p*
1	IGF1, ng/mL	89 [65–110]	120 [86–180]	100 [67–120]	1	0·79	0·16	0·03	<0·0001
2	PC aa C38:6	56 [42–66]	52 [47–70]	62 [55–72]	1	−0·40	−0·054	0·021	0·15
3	PC aa C40:6	25 [20–29]	22 [19–28]	25 [23−30]	1	−0·37	−0·064	0·021	0·1

Data are presented as median [IQR]. sPLS was conducted on log-transformed and standardized variables adjusted for age, sex, HIV and wealth. Metabolites were ranked in the top 10 at least 80% of the time across folds and having a > 0·3 PLS-component t_1_ loading value. Feature stability^1^ indicates the frequency at which the metabolite is selected in the top-10 with sparse PLS across 10-fold cross-validation. The loading^2^ value indicates the correlation strength of the metabolite with PLS-component t_1_. Associations between metabolites and height-for-age^3^ z-scores were tested using linear models on log transformed variables while adjusting for age, sex, HIV and wealth. Multiple testing correction was done based on all metabolites analysed using Benjamini & Hochberg's FDR. Significance threshold, FDR-corrected *p*-values <0·05. FDR, false discovery rate; sPLS, sparse partial least square analysis.

**Table 4:** Multivariate models exploring the relationship between IGF1, current stunting, and having survived childhood SAM.

Model 1: Current stunting in relation to IGF1				
		β (SE)	Partial R^2^	*p*
Height-for-age, z-scores		15·9 (3·5)	0·12	<0·0001
Age, months		1·3 (0·15)	0·34	<0·0001
Wealth		5·9 (2·7)	0·027	0·03
Sex	Female	Reference		
	Male	−35·3 (7·3)	0·14	<0·0001
HIV	Non-Reactive	Reference		
	Reactive	−21·0 (9·9)	0·028	0·04
	Unknown	6·4 (8·5)	–	0·45
Group	SAM survivor	–	–	–
	Control	–	–	–
Summary	Observations	148		
	Adjusted R^2^	0·489		
	Residual standard error	42·2		
	F Statistic	24·5 (df = 6; 141)		
	AIC	1536·3		

Model 2: Current stunting in relation to IGF1 and SAM survival compared to combined controls				
Height-for-age, z-scores		15·2 (3·5)	0·12	<0·0001
Age, months		1·2 (0·15)	0·33	<0·0001
Wealth		5·7 (2·6)	0·026	0·031
Sex	Female	Reference		
	Male	−34·6 (7·2)	0·14	<0·0001
HIV	Non-Reactive	Reference		
	Reactive	−15·1 (10·1)	0·002	0·14
	Unknown	0·46 (8·8)	–	0·96
Group	SAM survivor	Reference		
	Control	17·4 (7·7)	0·028	0·025
Summary	Observations	148		
	Adjusted R^2^	0·504		
	Residual standard error	41·6		
	F Statistic	22·3 (df = 7; 140)		
	AIC	1533		
	Model 2 vs. Model 1		*p* = 0·025	

Model 3: Current stunting in relation to IGF1 and SAM survival compared to community or sibling controls				
Height-for-age, z-scores		15·2 (3·5)	0·11	<0·0001
Age, months		1·2 (0·15)	0·31	<0·0001
Wealth		5·7 (2·6)	0·026	0·032
Sex	Female	Reference		
	Male	−34·2 (7·2)	0·13	<0·0001
HIV	Non-Reactive	Reference		
	Reactive	−15·1 (10·1)	0·002	0·14
	Unknown	−0·30 (8·8)	–	0·97
Group	SAM survivor	Reference		
	Sibling	21·9 (9·1)	0·027	0·018
	Community	13·1 (9·0)	–	0·15
Summary	Observations	148		
	Adjusted R^2^	0·503		
	Residual standard error	41·6		
	F Statistic	19·6 (df = 8; 139)		
	AIC	1534·1		
	Model 3 vs. Model 2		*p* = 0·36	

All multivariate models include adjustment for age, sex, HIV and wealth index. AIC; Akaike information criteria; β, regression coefficient; SE, standard error.

## 4 Discussion

Our study shows that, seven years post-treatment, children who survived early childhood SAM have similar metabolite profiles to those of sibling or community controls. Furthermore, there were no predictive signatures associated with the type of SAM experience (i.e., severe wasting vs. oedematous SAM) or with the severity of SAM during hospitalization. Current stunting was associated with low concentrations of IGF1 in both SAM and controls, and this relationship was modulated by having survived early childhood SAM.

### 4.1 SAM survivors *versus* controls

Previous studies assessing the metabolic profiles of children being treated for SAM have highlighted several changes in circulating metabolites compared to community controls [11,26]. While several metabolites recovered with treatment, some differences persisted even after achieving stabilization (e.g., low sphingomyelins and phosphatidylcholines). It was unclear if these persistent perturbations reflect a slow recovery of hepatic and/or gut function which go unresolved post-discharge [11], and could be mediated long term through epigenetic modification [27]. Indeed, SAM has been associated with both serious immediate consequences, including reductive adaptation, marked immunosuppression, and gut microbiota alterations, as well as long-term physiological consequences [28,29]. For example, a cohort study of Jamaicans (aged 17–50 years) who survived early childhood SAM showed higher glucose intolerance [30]; and we recently showed that our Malawian cohort of SAM survivors (aged 7–20 years) likely have a greater long-term risk of chronic disease, with greater stunting, and reduced lean mass, peripheral adiposity and muscle strength [14]. However, at this stage, these children had little indication of overt disease, as detected by blood pressure, lung function, cholesterol or glucose tolerance [14].

We report the first quantitative metabolomics study of long-term survivors of early childhood SAM, and shows that 7 years after treatment, baseline metabolic profiles of SAM survivors are similar to those of sibling and community controls. While this is reassuring, it is noteworthy that the controls, like the SAM survivors, live in settings with high levels of adversity – including stunting, morbidity and poor access to water and sanitation facilities. These conditions are associated with ill health, which could contribute to metabolic perturbations. For example, 20% of SAM survivors, 16% of siblings and 24% of community controls were hospitalized at least once for issues unrelated to SAM, and mean HAZ of community controls was −1·37 z-scores. In future studies, it will be useful to include a comparison of metabolic profiles of control children from higher socioeconomic status living in the same regions but with less exposure to adversity.

Taken together, these data indicate that early childhood SAM mainly impacts growth and muscle mass, rather than inducing persistent metabolic derangement that are detectable 7 years post-discharge. Early childhood SAM either: 1) impedes growth during a crucial period without long-term effects on epigenetics that regulate metabolism (i.e. occurs beyond the critical window known to have high sensitivity to epigenetic changes), or 2) induces subtle metabolic changes which only manifest through acute metabolic challenges or increased “load” induced by further aging or obesity [31].

### 4.2 Stunting and insulin-like growth factor I

In accord with previous studies [32,33], we showed that poor linear growth (stunting) was associated with lower circulating levels of IGF1, a central hormonal growth mediator. Also, this relationship is likely driven by a mechanism of growth hormone (GH) insensitivity since GH itself was not associated with either current stunting or SAM survival. Interestingly, a recent study following Zimbabwean infants from birth to 18 months found that levels of IGF1 were consistently lower among stunted infants from as early as 6 weeks of age [33]. However, this difference between stunted and non-stunted children was no longer apparent by 18 months. The authors hypothesized that IGF1 suppression by chronic, low-grade inflammation during fetal and postnatal life drives childhood stunting [34,35]. IGF1 can act directly on growth plates [35], and low levels can steadily slow linear growth during crucial developmental windows. SAM could impact the GH-IGF1 axis as, for a given level of stunting, SAM survivors had lower IGF1 compared to controls. Thus, either SAM survivors are slightly more sensitive to IGF1 growth maintenance or SAM induces a compensation mechanism that is independently able to maintain growth despite persistently low IGF1. We found that these relationships likely vary significantly depending on age and sex. Thus, understanding the impact of early childhood SAM on the GH-IGF1 axis would need to be better contextualized around crucial developmental windows such as puberty. Nonetheless, low IGF1 in early life is known to be associated with later risk of NCDs, particularly diabetes [36], and could mediate the hypothesized “thrifty phenotype” by regulating nutrition, growth and metabolism [37].

Interestingly, we found no other metabolites to be significantly associated with HAZ. This contrasts with a previous study comparing stunted and non-stunted children (aged 1–5 years) in Malawi which found that stunted children had significantly lower circulating levels of all nine essential amino acids, three conditional essential amino acids, and citrulline [38]. This may be explained by the generally low HAZ across our sample, including control children.

## 5 Strengths and limitations

The health and nutritional status of the control group is a possible study limitation since they are also stunted and have a history of poor health. Survivor bias should also be considered: SAM survivors are a select group as at least 46% of the original cohort have died since admission; half of these deaths occurred during admission and half since discharge; the majority of deaths were among children with HIV [24]. In addition, we have no record of birth weight which is also a known risk factor for developing NCDs in later life, and puberty onset was self-reported rather than clinically assessed. The selection of control children in the community did not always follow the systematic random strategy intended due to the limited availability of eligible children. The sample size limited further sub-group analysis or exploration of interactions. For example, it would have been interesting to study the effects of different timing of exposure to SAM. Lastly, our cohort of children were all admitted for inpatient treatment of SAM but treatment has since evolved and current standards for hospital admission differ.

There are however multiple strengths to our study including the deep phenotyping of SAM survivors from admission to 7-years post-discharge, the minimal loss-to-follow up, especially given the length of the follow-up period, and the use of both sibling and community controls.

## 6 Conclusion

In our cohort, SAM was not associated with detectable differences in baseline metabolism 7-years post-treatment, when compared to children from similar low socioeconomic communities. Measures of stunting in largely prepubescent children were associated with low IGF1 and, having experienced childhood SAM modulated this relationship. Low IGF1 in early life is known to be associated with later risk of NCDs, particularly diabetes, and could mediate the hypothesized “thrifty phenotype”. Future studies should consider whether SAM occurs beyond the critical window for epigenetic changes, thus largely affects growth, or whether metabolic dysregulations will only become apparent after cumulating a greater metabolic “load” due to modernized diets and aging.

## Funding sources

This research was funded by The Wellcome Trust through an “Enhancement Award” (grant number 101113/Z/13/A) and the Center for Healthy Active Kids, Hospital for Sick Children. GBG is a postdoctoral fellow of the Research Foundation Flanders (FWO).

The funder played no role in data collection, analysis, or interpretation, nor writing of the manuscript or the decision to submit it for publication. The corresponding author had full access to all the data and had final responsibility for the decision to submit for publication.

## Author contributions

NL, MK, MJN, and RB designed the research; NL, MK, PSD, EC, ET, MA and RB conducted data collection; CB, NL, DT, GBG, DW and RB analysed the data; CB, DT and NL wrote the manuscript and MJN had responsibility for final content. All authors read and approved the final manuscript.

## Declaration of Competing Interest

All authors declare no conflicts of interest.

## Appendix A Supplementary data

Supplementary material 1: Study Recruitment Flow Diagram; & Characteristics of children at hospital admission for severe acute malnutrition. Image 1

Suplementary material 2 Differences in metabolites between SAM survivors and siblings or community controls. Image 2
