# Supplementary material for: Metabolomics in plasma of Malawian children 7 years after surviving severe acute malnutrition: “ChroSAM” a cohort study
Source: eBioMedicine. 2019 Jun 27;45:464–72. doi: 10.1016/j.ebiom.2019.06.041 (PMC6642285; doi:10.1016/j.ebiom.2019.06.041)
Supplement: Supplementary file 1 — Supplementary material 1: Study Recruitment Flow Diagram; & Characteristics of children at hospital admission for severe acute malnutrition. [file mmc1.docx]

**Supplemental Material**

**Figure 1: Study Participant Flow Diagram throughout the cohort**


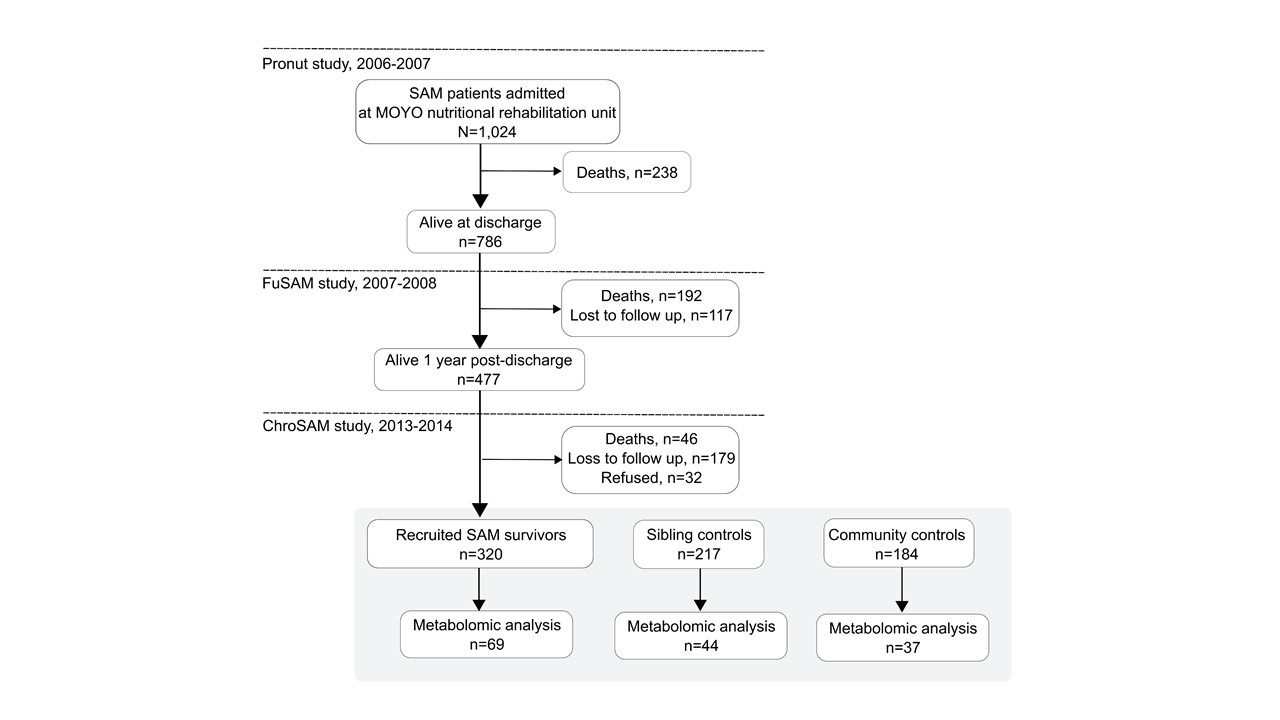


**Table 1: Characteristics of children at hospital admission for severe acute malnutrition**

|  | SAM survivors |
| --- | --- |
|  | n=69 |
| Female, n (%) | 30 (43%) |
| HIV reactive, n (%) | 21 (31%) |
| Kwashiorkor, n (%) | 55 (80%) |
| Age, months | 29.6 ± 19.2 |
| Length, cm | 78.5 ± 10.7 |
| MUAC*, cm | 10.5 ± 1.2 |
| Weight-for-age^¥^, z-score | -3.6 ± 1.3 |
| Height-for-age^¥^, z-score | -3.2 ± 1.3 |
| Weight-for-height^¥,Ϯ^, z-score | -2.9 ± 1.5 |
| BMI-for-age^¥^, z-score | -2.4 ± 1.6 |
| Duration of hospital stay, days | 8.8 ± 4.6 |
|  |  |

Data are presented as number and percentage, or averages and standard deviation. *MUAC measures only include children without edema.¥ The lowest weight recorded during hospital admission was used to determine anthropometric z-scores. ϮWeight-for-height is calculated only in children under 5 years of age.

**Table 2: See Excel sheet**
